# The Book World of Medicine and Science

**Published:** 1896-02-22

**Authors:** 


### The Book World of Medicine and Science.

Cottage Hospitals, General, Fever, and Convalescent: Their Progress, Management, and Work in Great Britain and Ireland and the United States of America, with many plans and illustrations. By Henry C. Burdett. (London: The Scientific Press, Limited, 428, Strand, W.C. 1896. Price 10s. 6d.)

The book before us is especially written for the guidance and assistance of those concerned in founding, building, and administrating cottage hospitals. The information given is as complete as it is possible to render it within the compass of one work, and the whole aspect is ably and practically dealt with by Mr. Burdett, whose unique experience renders him especially fitted to deal with the subject. From the foundation to the completion of a cottage hospital information and guidance is given for every stage of the undertaking. The book is provided with numerous plans, illustrations, and descriptions of existing institutions. Their attendant advantages and disadvantages are pointed out, and model plans are supplied, designed by the author. Thus founders and architects of cottage hospitals will be enabled in the future to avoid material mistakes in construction, and to secure well-arranged and economical buildings. Site, cost, drainage, and water supply are fully discussed. For the conduct of the institution, rules are given which have proved useful in practice for the medical, administrative, nursing, and domestic departments. Model reports, a system of accounts, and other matters are provided for the secretariat. The lists of medical appliances, furniture, and fittings will be found most useful. The volume closes with an alphabetical list of existing cottage hospitals. The book will be found indispensable to those concerned in the establishment of these institutions, and it is not too much to say that everyone interested in cottage hospitals should obtain a copy. Certainly no hospital or architect's library can be regarded as complete without it, and thanks should be tendered to the author for placing so invaluable a work within the reach of all interested in the subject. The book has been practically re-written. During the fifteen years which have elapsed since the second edition was issued cottage hospitals have become an important part of our hospital system, and the preliminary chapters which deal with their origin and growth will be found especially interesting. Experience shows these small hospitals to be almost an unqualified success. Their hygienic and moral teaching has been excellent, and as regards expenditure, they show to advantage in comparison with larger institutions. The whole subject is a most instructive study, and we refer our readers to the ably-written pages of the present volume.

Inorganic Chemistry. Blackie's Science Text-books. By A. Humboldt Sexton. Fourth edition, revised and enlarged. (London: Blackie and Son. 1895. 360 pp. 2s. 6d.)

The appearance of a new edition of this little work will no doubt be hailed with pleasure by a large number of students who are preparing for the examinations of the Science and Art Department. Heretofore it has closely kept pace with the requirements of the Kensington syllabus, and in this, the fourth edition, we notice several modifications in conformity with the changes and additions which have lately been made by the department. As is well known, Professor Sexton combines in his book notes for practical experimentation illustrative of the subject matter contained in the text; in this respect his work differs little from many others on the same elementary subject. Where it does differ, however, is in the handling of the physical aspect of the subject. Not only are the physical properties of chemical bodies fully described, but the borderland and connecting links between the two subjects—chemistry and physics—receive that full attention which, to the confusion of the student, is generally omitted in independent text-books on both these branches of natural philosophy. Applied mathematics also form a conspicuous feature of the edition, including problems in quantitative analysis, the calculation of formulæ, percentage composition, volume of gases, and on those other questions which go to make up the subject of chemical arithmetic. This should be a great help to students, as it is in this side of the subject that students most often fail to satisfy the examiners. This new edition of Professor Sexton's book will doubtless secure for the work an acceptance in the future no less successful than that which has marked its history in the past.

